# The small RNA SgrS: roles in metabolism and pathogenesis of enteric bacteria

**DOI:** 10.3389/fcimb.2014.00061

**Published:** 2014-05-08

**Authors:** Maksym Bobrovskyy, Carin K. Vanderpool

**Affiliations:** Department of Microbiology, University of Illinois at Urbana-ChampaignUrbana, IL, USA

**Keywords:** small RNA, SgrR, PtsG, glucose-phosphate stress, glycolysis, phosphoenolpyruvate phosphotransferase system

## Abstract

Bacteria adapt to ever-changing habitats through specific responses to internal and external stimuli that result in changes in gene regulation and metabolism. One internal metabolic cue affecting such changes in *Escherichia coli* and related enteric species is cytoplasmic accumulation of phosphorylated sugars such as glucose-6-phosphate or the non-metabolizable analog α-methylglucoside-6-phosphate. This “glucose-phosphate stress” triggers a dedicated stress response in γ-proteobacteria including several enteric pathogens. The major effector of this stress response is a small RNA (sRNA), SgrS. In *E. coli* and *Salmonella*, SgrS regulates numerous mRNA targets via base pairing interactions that result in alterations in mRNA translation and stability. Regulation of target mRNAs allows cells to reduce import of additional sugars and increase sugar efflux. SgrS is an unusual sRNA in that it also encodes a small protein, SgrT, which inhibits activity of the major glucose transporter. The two functions of SgrS, base pairing and production of SgrT, reduce accumulation of phosphorylated sugars and thereby relieve stress and promote growth. Examination of SgrS homologs in many enteric species suggests that SgrS has evolved to regulate distinct targets in different organisms. For example, in *Salmonella*, SgrS base pairs with *sopD* mRNA and represses production of the encoded effector protein, suggesting that SgrS may have a specific role in the pathogenesis of some γ-proteobacteria. In this review, we outline molecular mechanisms involved in SgrS regulation of its target mRNAs. We also discuss the response to glucose-phosphate stress in terms of its impact on metabolism, growth physiology, and pathogenesis.

Over the last decade, small RNAs have emerged from relative obscurity to take their places as central regulators of gene expression in organisms from all three domains of life. While hundreds of small RNAs in dozens of bacterial genomes have been identified by computational or experimental methods, the functions of the vast majority of these remain a mystery. Nevertheless, we have learned a great deal about a small number of “model” bacterial sRNAs, including how their production is regulated, what targets they in turn regulate and the physiological outcomes of sRNA-mediated regulation. In this review, we first provide a brief overview of features of regulatory sRNAs that act on mRNAs through base pairing interactions. We then focus on one well-characterized sRNA, SgrS (sugar transport related sRNA) and describe its activities on target mRNAs and how these activities regulate bacterial metabolism and pathogenesis.

## Mechanisms of regulation by bacterial small RNAs

### Basic characteristics of sRNA-mediated regulation

Several mechanistic classes of sRNAs have been identified in diverse bacterial species. Many characterized sRNAs act by base pairing with mRNA targets to control mRNA stability and translation. Such sRNAs are often between 50 and 300 nucleotides in length and require an RNA chaperone, Hfq, for their stability and regulatory effects on target mRNAs (Sledjeski et al., 2001; Moller et al., 2002; Zhang et al., 2002). Hfq is a hexameric ring protein with sRNA- and mRNA-binding faces (Mikulecky et al., 2004; Link et al., 2009; Zhang et al., 2013). On sRNAs, Hfq binds to stem-loop terminator structures preceded by A/U rich sequences (Valentin-Hansen et al., 2004; Otaka et al., 2011; Ishikawa et al., 2012). Hfq-binding sites are located in 5' untranslated regions (UTRs) of many mRNAs that are regulated by sRNAs (Soper and Woodson, 2008; Link et al., 2009; Salim and Feig, 2010; Salim et al., 2012). Binding of Hfq to both sRNAs and mRNAs increases their local concentrations, stimulates structural remodeling to facilitate pairing and increases annealing rates of cognate pairs (Fender et al., 2010; Maki et al., 2010; Soper et al., 2010; Hopkins et al., 2011). Other factors involved in sRNA-mRNA regulatory transactions include RNase E and components of the degradosome (Masse and Gottesman, 2002; Masse et al., 2003; Morita et al., 2005). Polynucleotide phosphorylase (PNPase, a 3'–5' exonuclease), RhlB helicase and enolase assemble on RNase E to form a degradosome complex required for bulk mRNA turnover in proteobacteria (Carpousis, 2007). RNase E binds A/U-rich single-stranded regions of RNAs and is responsible for sRNA-mediated mRNA degradation (Carpousis et al., 2009; Belasco, 2010; Prevost et al., 2011).

### Repression by sRNAs

The canonical mechanism of negative regulation by sRNAs involves base pairing interactions that directly inhibit translation initiation because the sRNA sequesters mRNA sequences required for stable ribosome binding, in the region from ~20 nt upstream to 20 nt downstream of the start codon (Beyer et al., 1994; Huttenhofer and Noller, 1994). As more detailed studies of sRNA regulatory mechanisms have been performed, variations on this theme have been discovered. Binding of sRNAs to mRNA sequences outside the region recognized by the ribosome can still inhibit translation initiation (Darfeuille et al., 2007; Sharma et al., 2007; Bouvier et al., 2008; Holmqvist et al., 2010), for example, by recruiting Hfq to bind at a site overlapping the ribosome binding site (RBS) (Desnoyers and Masse, 2012) or by sequestering mRNA sequences that apparently act as translational enhancer elements (Bandyra et al., 2012; Desnoyers et al., 2013).

Regardless of the mechanism, sRNA-mediated translational repression is often coupled to mRNA degradation by an RNase E degradosome-dependent pathway. Translating ribosomes protect mRNA from RNase E degradation (Dreyfus, 2009), thus sRNA inhibition of translation unmasks RNase E recognition sites and makes target mRNAs susceptible to degradation (Prevost et al., 2011). Though translational repression and mRNA degradation are typically coupled, this is usually not obligatory for gene silencing. In other words, mutations that abrogate RNase E-dependent turnover of sRNA targets have no impact on translational repression (Morita et al., 2006; Rice and Vanderpool, 2011; Rice et al., 2012). While less commonly described, in some cases sRNA-mediated translational repression has no significant effect on mRNA turnover (Moller et al., 2002). Conversely, there are a handful of known cases where sRNAs do not directly affect translation but rather specifically target mRNAs for degradation (Desnoyers et al., 2009; Pfeiffer et al., 2009).

### Activation by sRNAs

sRNAs can also activate gene expression post-transcriptionally. Positive regulation of target mRNAs is achieved via activation of translation or stabilization of the target transcript. Some mRNAs have intrinsic secondary structures in their 5' UTRs that hinder translation, for example, because the RBS is sequestered. Binding of sRNAs to these 5' UTRs can prevent formation of translation-inhibitory secondary structures and therefore activate translation (Morfeldt et al., 1995; Lease et al., 1998; Prevost et al., 2007). Another mechanism of activation by sRNAs involves sRNA-mRNA base pairing that alters accessibility of RNase E recognition sites on mRNA targets. sRNA-mRNA binding can induce cleavage of a target transcript, resulting in a processed mRNA with an accessible RBS (Obana et al., 2010) or alternatively sRNA base pairing with an intrinsically unstable mRNA can occlude an RNase E recognition site and prevent cleavage (Papenfort et al., 2013).

## The sRNA SgrS mediates the response to glucose-phosphate stress

### Characteristics of SgrS

**Figure 1:**
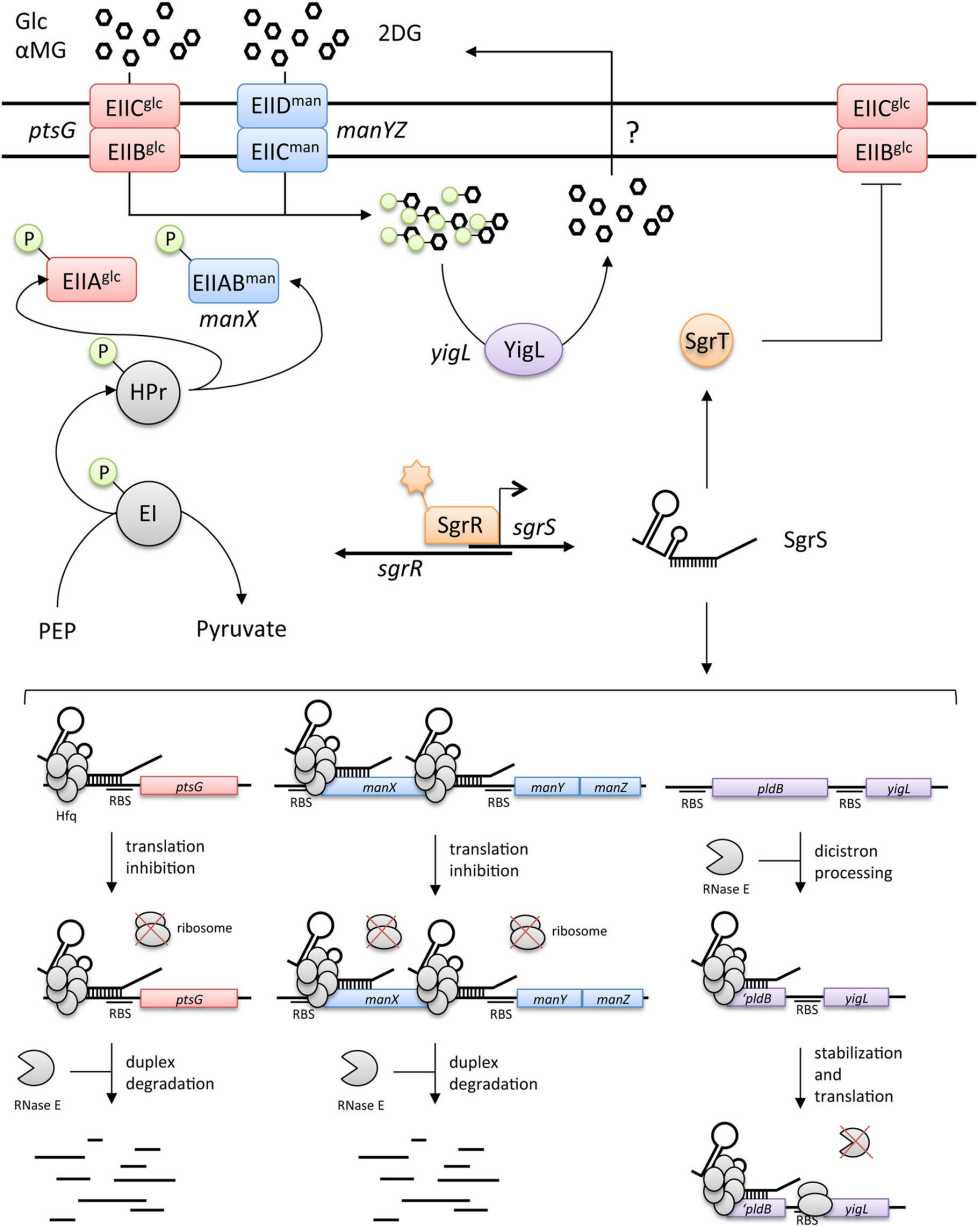
**Current model for the role of SgrS in the glucose-phosphate stress response**. The top panel illustrates the main features of the phosphoenolpyruvate phosphotransferase system (PTS), which transports numerous carbohydrates as well as glucose analogs (αMG, 2DG: α-methyl glucoside and 2-deoxy glucose, respectively). Glucose-phosphate stress is associated with accumulation of sugar-phosphates (hexagons with attached green circles). The stress response is initiated by the activated transcription factor, SgrR, which induces SgrS synthesis. SgrS has two functions; the first is base pairing-dependent regulation of target mRNAs (illustrated in lower panel), the second is production of the ~40 amino acid protein SgrT. SgrT acts to repress activity of the EIICB^glc^ (PtsG) transporter (top panel). The base pairing activity results in repression of two mRNA targets encoding PTS sugar transporters, *ptsG* and *manXYZ*, and activation of a third mRNA target encoding a phosphatase, *yigL* (described in detail in the text). Altogether, the base pairing activity of SgrS on these various targets inhibits further uptake of sugar-phosphates by inhibiting production of sugar transporters and promotes sugar efflux by providing neutral sugar substrates that are pumped out by an unknown efflux pump (indicated by a “?”).

**Figure 2:**
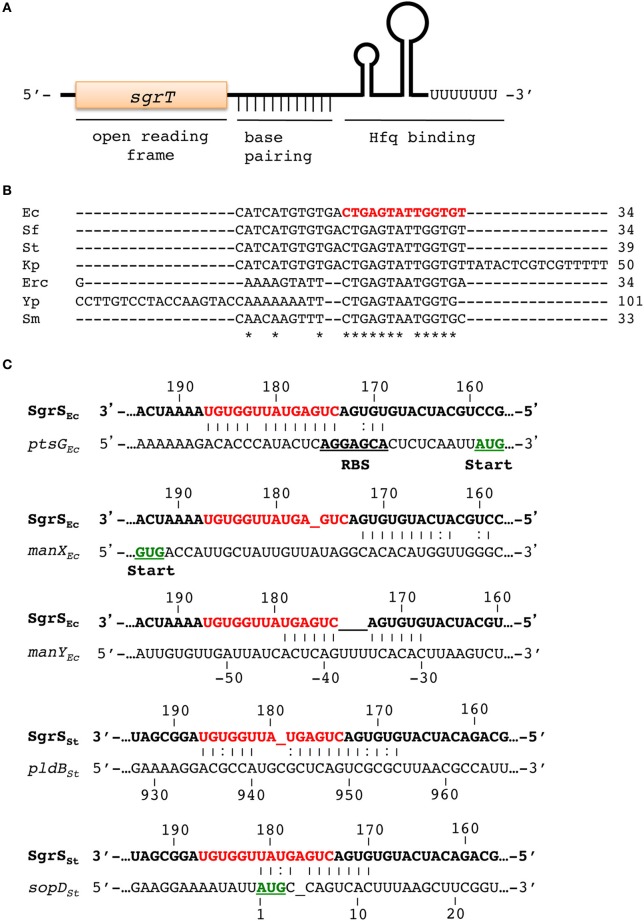
**Characteristics of SgrS and SgrS-mRNA base pairing interactions**. **(A)** The main functional domains of SgrS are illustrated. The *sgrT* open reading frame is located at the 5' end, the conserved base pairing region is downstream of *sgrT* and upstream of the intrinsic terminator hairpin (which comprises the Hfq-binding domain). **(B)** Alignment of the base pairing region of SgrS homologs from enteric species. The most conserved region is indicated by asterisks below the alignment and in red for the *E. coli* (Ec) homolog. Abbreviations for other species: Sf, *Shigella flexneri*; St, *Salmonella enterica* serovar Typhimurium; Kp, *Klebsiella pneumoniae*; Erc, *Erwinia carotovora*; Yp, *Yersinia pestis*; Sm, *Serratia marcescens*. **(C)** SgrS-mRNA base pairing interactions. Interactions with each confirmed SgrS target are shown (species abbreviations are as in **B**). Watson-Crick base interactions G-C and A-U are indicated with vertical lines and non-canonical G-U pairs are denoted with two dots. The conserved SgrS base pairing region is indicated in red. Start codons are indicated in green and underlined. Ribosome binding sites (RBS) are bold and underlined.

SgrS is a 227-nt (in *E. coli*) Hfq-binding sRNA (Zhang et al., 2003) that is produced during “glucose-phosphate stress,” which is characterized by accumulation of phosphosugars like glucose-6-phosphate (G6P) or its analog α-methyl glucoside-6-phosphate (αMG6P) (Vanderpool and Gottesman, 2004; Wadler and Vanderpool, 2007). Glucose and α-methyl glucoside (αMG) are taken up and phosphorylated by the phosphoenolpyruvate phosphotransferase system (PTS) (Postma et al., 1993; Deutscher et al., 2006; Gorke and Stulke, 2008). If metabolism of G6P is blocked (e.g., by mutation of genes encoding early glycolytic enzymes), or if cells accumulate non-metabolizable αMG6P, *sgrS* is induced by the transcription factor SgrR (Figure 1) (Vanderpool and Gottesman, 2007). Both SgrS and SgrR are essential for cell growth under glucose-phosphate stress conditions (Vanderpool and Gottesman, 2004). SgrS regulates a number of mRNA targets through base pairing interactions involving a conserved region near the 3' end (Figure 2A, conserved residues are in red in Figure 2B). In addition, the 5' end encodes a 43-amino-acid protein called SgrT (Figure 2A). Remarkably, SgrS base pairing activity and SgrT function by independent regulatory mechanisms to allow cells to cope with glucose-phosphate stress and continue growing (Figure 1) (Wadler and Vanderpool, 2007; Balasubramanian and Vanderpool, 2013).

### Targets of SgrS regulation

EIICB^glc^, encoded by *ptsG*, mediates transport and phosphorylation of glucose and αMG. SgrS negatively regulates *ptsG* by directly inhibiting *ptsG* translation initiation by base pairing with the *ptsG* 5' UTR near the RBS (Figures 1, 2C). SgrS-dependent translational repression requires Hfq and stimulates *ptsG* mRNA degradation by an RNase E-dependent pathway (Vanderpool and Gottesman, 2004; Kawamoto et al., 2006; Maki et al., 2008). SgrS is highly unstable in the *hfq* mutant strain, highlighting the essential role of Hfq in SgrS-dependent regulation (Balasubramanian and Vanderpool, 2013).

SgrS also represses *manXYZ*, which encodes a PTS transporter of relatively broad substrate specificity. SgrS repression of *manXYZ* is carried out through a more complex mechanism compared to *ptsG*. Two distinct SgrS binding sites on *manXYZ* mRNA are located within early *manX* coding sequences and within the *manX-manY* intergenic region (Figures 1, 2C). SgrS binding at the *manX* site is responsible for translational repression of *manX*, but has no effect on translation of *manY* or *manZ* (Rice et al., 2012). The intergenic SgrS binding inhibits translation of *manY* and *manZ* (translation of *manY* and *manZ* is coupled) and this is independent of *manX* regulation (Rice et al., 2012). SgrS binding at each site individually does not affect *manXYZ* mRNA stability; pairing at both sites is required for RNase E-dependent degradation of *manXYZ* mRNA (Rice et al., 2012).

A third SgrS target, *yigL* mRNA, which encodes a haloacid dehalogenase-like phosphatase (Koonin and Tatusov, 1994), is positively regulated by SgrS (Papenfort et al., 2013). Synthesis of YigL is induced by SgrS in response to glucose-phosphate stress. The *yigL* gene is in an operon with the upstream *pldB*, however SgrS activates only *yigL*. In the absence of SgrS, RNase E processing of the *pldB-yigL* transcript yields an mRNA that is susceptible to further degradation. When SgrS is produced, it base pairs with a sequence on the processed '*pldB-yigL* mRNA (Figures 1, 2C) and prevents further degradation. This mechanism of positive regulation is unusual in that an initial processing event is required to allow SgrS access to its binding site because cleavage within *pldB* frees the *pldB* region from translating ribosomes (Papenfort et al., 2013). Moreover, activation of *yigL* by SgrS is translation-independent. SgrS stabilizes *yigL* mRNA by occluding a specific RNase E cleavage site upstream of the *yigL* coding region, not by enhancing *yigL* translation (Papenfort et al., 2013). A similar translation-independent mechanism of mRNA stabilization was recently described for the RydC sRNA-*cfa* mRNA regulatory pair (Frohlich et al., 2013).

### Glucose-phosphate stress physiology

Targets of SgrS include sugar transporters and a sugar phosphatase. SgrS-mediated repression of sugar transporters diminishes cells' capacity to take up sugars and therefore reduces further phosphosugar accumulation (Figure 1). However, this effect is not immediate: PtsG protein has a half-life of ~80 min (Papenfort et al., 2013), so merely stopping new synthesis of PtsG would not provide a fast remedy for the problem of phosphosugar accumulation. The activation of YigL synthesis by SgrS addresses this problem since dephosphorylation of sugars allows their efflux (Figure 1) (Winkler, 1971; Haguenauer and Kepes, 1972; Papenfort et al., 2013). Growth competition experiments between wild-type and *sgrS* mutants provided insight into how regulation of different SgrS targets contributes to stress resistance and growth during glucose-phosphate stress (Sun and Vanderpool, 2013). When cells are stressed while growing in rich medium, SgrS-mediated regulation of *ptsG* mRNA alone is sufficient to confer wild-type levels of growth. In contrast, cells stressed in minimal media are far more growth inhibited, and repression of *ptsG* alone is not sufficient to rescue growth. In minimal media stress conditions, repression of *ptsG* and activation of *yigL* are necessary, but not sufficient for full growth rescue (Sun and Vanderpool, 2013). These findings illustrate the poorly understood influence of nutrient availability on the severity of glucose-phosphate stress. Moreover, these results highlight the fact that additional unknown SgrS targets are involved in the stress response.

Phosphosugar intermediates of central metabolism provide precursors for biomass and energy, yet, as illustrated by glucose-phosphate stress, excessive accumulation of phosphosugars is detrimental to cell growth. Other types of phosphosugar stress also cause growth inhibition or cell lysis (Yarmolinsky et al., 1959; Englesberg et al., 1962; Irani and Maitra, 1977; Lee et al., 2009). In most cases, the mechanisms responsible for phosphosugar-associated inhibition or lysis have not been defined. However, recent work suggests that in some cases phosphosugars themselves are not directly inhibitory. Rather, accumulation of phosphosugars is accompanied by depletion of other metabolites, and stress is ameliorated by supplementation with the limiting metabolites (Lee et al., 2009, 2013; Richards et al., 2013). Glucose-phosphate stress is so far associated with accumulation of a few sugar-phosphate intermediates of upper glycolysis (Morita et al., 2003; Vanderpool and Gottesman, 2004; Sun and Vanderpool, 2013). A recent study implicates depletion of intermediates of lower glycolysis, particularly phosphoenolpyruvate (PEP) as an important cause of glucose-phosphate stress. When αMG is taken up and phosphorylated, it cannot be metabolized to replenish glycolytic intermediates. Thus, PEP utilized to drive αMG uptake is not replaced via glycolytic metabolism. Under these conditions, SgrS regulation of target mRNAs and production of SgrT limits PEP consumption by reducing levels and activity of PtsG (Figure 1). In *sgrS* mutants, exposure to αMG results in strong growth inhibition (Vanderpool and Gottesman, 2004; Richards et al., 2013) that is largely reversed by supplementing stressed cultures with glycolytic intermediates (Richards et al., 2013). The ratios of PEP to pyruvate seem to be particularly relevant for growth during glucose-phosphate stress. Increasing pyruvate levels during stress results in lysis of *sgrS* mutant cells, whereas increasing PEP levels rescues cell growth (Richards et al., 2013). The observation that stress (and growth inhibition) is more severe when cells are growing in minimal compared to rich media is also consistent with metabolite depletion as an underlying cause of glucose-phosphate stress. In rich media, cells do not have to synthesize many biosynthetic intermediates. In contrast, growth in minimal media requires *de novo* biosynthesis of amino acids. Thus, depletion of glycolytic intermediates during glucose-phosphate stress would have more severe effects on growth under conditions where these same intermediates are needed as precursors for biosynthesis. Consistent with this idea, supplementation of minimal media with amino acids improves stress recovery in minimal medium (Sun and Vanderpool, 2013).

The transcription factor SgrR also plays an important, but not fully characterized role in glucose-phosphate stress physiology. SgrR activates expression of *sgrS* and at least two other genes during glucose-phosphate stress: *setA*, encoding an efflux pump (Liu et al., 1999; Sun and Vanderpool, 2011), and *alaC* (formerly *yfdZ*), a glutamate-pyruvate aminotransferase (Vanderpool and Gottesman, 2007; Kim et al., 2010). The role of *alaC* in helping cells recover from glucose-phosphate stress is unknown. In contrast, *setA*, which is encoded just downstream of *sgrS*, is important for growth recovery under certain stress conditions (Sun and Vanderpool, 2011). Given its function as an efflux pump, the hypothesis that SetA was responsible for export of αMG was tested, but was not supported (Sun and Vanderpool, 2011). Thus, the role of SetA in glucose-phosphate stress also remains elusive.

## Distribution and function of SgrS in γ-proteobacteria

### Identification of SgrS in enteric bacteria

SgrS homologs were identified in many γ-Proteobacteria, including *Escherichia* sp., *Salmonella* sp., *Shigella* sp., *Yersinia* sp, *Serratia* sp., *Klebsiella pneumoniae* and *Erwinia* sp. (Horler and Vanderpool, 2009). The *sgrR-sgrS* intergenic region (containing the *sgrS* promoter) is highly conserved, suggesting that SgrR regulates *sgrS* expression in all these organisms. All identified SgrS homologs contain a Rho-independent terminator and most possess an additional stem-loop structure upstream of the terminator; these two structures are important for Hfq binding to SgrS (Figure 2A) (Horler and Vanderpool, 2009; Otaka et al., 2011; Ishikawa et al., 2012). While the overall conservation of SgrS is low, a short stretch of ~13 nts near the SgrS 3' end is nearly invariant (Figure 2B). This SgrS sequence is complementary to the translation initiation regions of *ptsG* mRNAs in all species where an SgrS homolog was found (Horler and Vanderpool, 2009). Mutation of residues G176 and G178 within the conserved region of *E. coli* SgrS abrogates SgrS-mediated repression of *ptsG* mRNA and prevents recovery from glucose-phosphate stress (Maki et al., 2008). Introduction of analogous mutations in the conserved regions of SgrS homologs from *Salmonella, E. carotovora, Y. pestis* and *K. pneumoniae* similarly prevented regulation of *ptsG* (Wadler and Vanderpool, 2009). Regulation of other targets is less well conserved among SgrS homologs. The SgrS sequences required for base pairing with *manX* are upstream of the conserved region (Figure 2C) and are poorly conserved among SgrS homologs. SgrS homologs from *Salmonella* and *K. pneumoniae* have the same predicted SgrS-*manX* base pairing interaction and *manX* translation is regulated as expected. In contrast, *E. carotovora* and *Y. pestis* SgrS homologs have changes in the *manX* pairing site resulting in loss of complementarity to their cognate *manX* and were accordingly shown not to regulate *manX* translation (Rice and Vanderpool, 2011).

### Conservation of SgrT

While the exact molecular function of SgrT has not been reported, available data strongly suggest that this small protein interacts directly with PtsG protein to inhibit its activity (Wadler and Vanderpool, 2007). Most SgrS homologs contain open reading frames similar in size to *E. coli* SgrT (~40 amino acids) (Horler and Vanderpool, 2009). While the primary amino acid sequence of putative SgrT homologs was not well conserved, homologs from *Salmonella, Klebsiella*, and *Erwinia* were functional when expressed in an *E. coli sgrST* mutant (Wadler and Vanderpool, 2009). Interestingly, some species with SgrS homologs appear to lack a functional SgrT. In *Yersinia* sp., SgrS appears to be truncated at the 5' end, and SgrS from *Yersinia* species ranges in size from ~85 to 140 nt and lacks the *sgrT* open reading frame. In pathogenic *E. coli* O157:H7 strains, a point mutation in the SgrS 5' region alters the *sgrT* start codon, presumably abrogating SgrT production in these strains.

Differential presence and absence of SgrT in organisms that possess SgrS led to a closer comparison of *E. coli* K12 and *Salmonella* SgrS sRNAs. In *E. coli* K12, *sgrT* alone (without the region of SgrS involved in base pairing with mRNAs) was not sufficient to allow growth rescue during glucose-phosphate stress conditions. This is in part due to very low levels of SgrT produced from the native *sgrS* allele in *E. coli* (Wadler and Vanderpool, 2009)*. E. coli* SgrS has a sequence in the 5' region that forms a structure that inhibits *sgrT* translation. On the other hand, *Salmonella* SgrS does not have the same inhibitory structure and therefore produces more SgrT than *E. coli* SgrS (Wadler and Vanderpool, 2009; Balasubramanian and Vanderpool, 2013). While native levels of SgrT production have not been investigated in *Erwinia* or *Klebsiella* species, it was observed that ectopic production of SgrT homologs from these organisms in an *E. coli sgrST* mutant rescued growth during glucose-phosphate stress (Wadler and Vanderpool, 2009). Thus, SgrT is functionally conserved when it is present, but levels of SgrT production vary among bacteria.

### SgrS regulation of sopD mRNA

Although SgrS is conserved among enteric bacteria, divergence in primary sequence has resulted in species-specific target regulons, exemplified by the finding that *Erwinia* and *Yersinia* SgrS homologs do not regulate their cognate *manXYZ* homologs (Rice and Vanderpool, 2011). Another instance of species-specific regulation by SgrS is regulation of the *Salmonella*-specific gene *sopD* (Papenfort et al., 2012). SopD is an effector delivered to host cells through the Type 3 Secretion Systems (T3SSs) encoded on *Salmonella* pathogenicity island (SPI)-1 and SPI-2 (Brumell et al., 2003) and it functions as a general virulence factor in mice (Jiang et al., 2004; Bakowski et al., 2007). Regulation of *sopD* by SgrS involves base pairing interactions between the conserved region of SgrS and the early coding sequence of *sopD* mRNA (Figure 2C); the interaction inhibits translation initiation and stimulates *sopD* mRNA degradation (Papenfort et al., 2012). Interestingly, *Salmonella* encodes a second SopD protein, SopD2, which shares 42% identity with SopD and likely arose from a duplication (Jiang et al., 2004). The predicted SgrS-*sopD2* base pairing interaction differs from SgrS-*sopD* at only a single position, a wobble G:U base pair instead of the G:C base pair. Remarkably, this interaction that differs by only a single hydrogen bond prevents regulation of *sopD2* by SgrS (Papenfort et al., 2012).

While the biological significance of *sopD* regulation by SgrS is not yet clear, the inclusion of *sopD* in the *Salmonella* SgrS regulon illustrates plasticity in the evolution of sRNA regulons. The presence of *sgrR-sgrS-sgrT* in the same genomic context in pathogenic and non-pathogenic γ-proteobacteria (Horler and Vanderpool, 2009) suggests that this is an ancestral, or “core” RNA among these organisms. Yet, this core sRNA has acquired the ability to regulate a gene that was horizontally acquired by *Salmonella*. Studies of other SgrS homologs in pathogenic and non-pathogenic enteric bacteria will surely shed light on the breadth of regulatory activities of this fascinating dual-function sRNA.

### Conflict of interest statement

The authors declare that the research was conducted in the absence of any commercial or financial relationships that could be construed as a potential conflict of interest.
